# Genomic Investigation into Strain Heterogeneity and Pathogenic Potential of the Emerging Gastrointestinal Pathogen *Campylobacter ureolyticus*

**DOI:** 10.1371/journal.pone.0071515

**Published:** 2013-08-30

**Authors:** Susan Bullman, Alan Lucid, Daniel Corcoran, Roy D. Sleator, Brigid Lucey

**Affiliations:** 1 Department of Biological Sciences, Cork Institute of Technology, Cork, Ireland; 2 Department of Medical Microbiology, Cork University Hospital, Cork, Ireland; Quuen's University Belfast, United Kingdom

## Abstract

The recent detection and isolation of *C. ureolyticus* from patients with diarrhoeal illness and inflammatory bowel diseases warrants further investigation into its role as an emerging pathogen of the human gastrointestinal tract. Regarding the pathogenic mechanisms employed by this species we provide the first whole genome analysis of two *C. ureolyticus* isolates including the type strain. Comparative analysis, subtractive hybridisation and gene ontology searches against other *Campylobacter* species identifies the high degree of heterogenicity between *C. ureolyticus* isolates, in addition to the identification of 106 putative virulence associated factors, 52 of which are predicted to be secreted. Such factors encompass each of the known virulence tactics of pathogenic *Campylobacter* spp. including adhesion and colonisation (CadF, PEB1, IcmF and FlpA), invasion (*ciaB* and 16 *virB-virD4* genes) and toxin production (S-layer RTX and ZOT). Herein, we provide the first virulence catalogue for *C. ureolyticus*, the components of which theoretically provide this emerging species with sufficient arsenal to establish pathology.

## Introduction

Within the last decade a growing number of atypical *Campylobacter* species have been reported as emerging human pathogens [1]. Traditionally, *C. jejuni* and *C. coli* have been the main species associated with human illness, however advances in molecular diagnostics coupled with the development of novel culture techniques have facilitated the detection and isolation of a range of under reported and highly fastidious *Campylobacter* species [2], [3], including *C. concisus* and more recently *C. ureolyticus* [4], [5].

*C. ureolyticus* (previously *Bacteroides ureolyticus*) has only recently been classified within the *Campylobacter* genus [6]. Although in 1991, Vandamme et al. proposed that *B. ureolyticus* be reclassified as a member of the *Campylobacter* genus [7], its fatty acid profile and hydrolysis of gelatin and casein differentiated this organism from other *Campylobacter* species and *B. ureolyticus* remained as ‘species *incertae sedis’* [6], [8], [9]. Almost two decades later, employing a polypahsic approach, Vandamme and colleagues reported that *B. ureolyticus* shared (i) respiratory quinone content, (ii) DNA base ratio, and (iii) phenotypic characteristics with *Campylobacter* species, including *Campylobacter jejuni* and resulted in the reclassification of *Bacteroides ureolyticus* as *Campylobacter ureolyticus* [6].

Historically, *C. ureolyticus* has been associated with a range of diseases, including superficial ulcers, gangrenous lesions, nongonococcal urethritis, bacterial vaginosis, and of late, male infertility [6], [10], [11], [12]. Furthermore, analogous to several other emerging and atypical *Campylobacter* species, *C. ureolyticus* has been linked with periodontal lesions, including gingivitis and peridontitis [2], [13], [14].

Recent work has led to the detection and subsequent isolation of *C. ureolyticus* as the sole pathogen from faecal samples of diarrheic patients [4], [15], [16]. Using a species specific PCR (targeting the *hsp60* gene), *C. ureolyticus* is now believed to be the second most common *Campylobacter* species detected in diarrhoeic patients surpassing the established pathogen *C. coli* and exceeded only by *C. jejuni* [13]. Additionally, analysis of infectivity data reveals a predominance of *C. ureolyticus* in patients at extremes of age (<5 years and >70 years) suggesting an opportunistic nature for the pathogen [17]. Furthermore, we have noted a seasonal prevalence and have identified potential reservoirs of infection [18].

Following our initial report, *C. ureolyticus* has been detected at significantly higher rates in patients with Ulcerative Colitis (21.7%) in comparison to healthy controls (3.1%) [19]. In support of this, a New South Wales study [20], report the detection and isolation of *C. ureolyticus* from biopsy specimens and faecal samples from children with newly diagnosed Crohn's disease (CD). This group later report on the pathogenic potential of *C. ureolyticus:* observing that their strain *C. ureolyticus* UNSWCD was capable of colonizing and adhering to intestinal cells - resulting in cellular damage and microvillus degradation [21]. As such, the recent emergence of *C. ureolyticus* in patients with gastrointestinal illness, at higher levels than the healthy controls, provides a compelling case that *C. ureolyticus* is likely to be an emerging gastrointestinal pathogen of some importance.

Despite the growing evidence to suggest that non-*C. jejuni*/*C. coli* species are significant contributors to human disease [2], [15], [22], our existing understanding of *Campylobacter* pathogenesis is essentially restricted to *C. jejuni*. Furthermore, the literature regarding the mechanisms of *C. jejuni* invasion is highly controversial, whereby some groups report the paracellular route and others described the transcellular model or a mix of both [23], [24], [25], [26], [27]. In general, the past decade has provided us with substantial findings, revealing many of the virulence components of *C. jejuni* [28], however the exact mechanism of its pathology is as yet still unclear [29], [30]. The small, curved shape of this Gram-negative bacterium, coupled with flagella-mediated motility, allow *C. jejuni* to penetrate intestinal mucus [31], where it can then adhere to epithelial cells via various surface associated adhesions, such as CadF and FlpA, which mediate binding to host tissue fibronectin [32]. Once attached, the bacterium then employs a range of secretion systems including the flagellar type III, the type IV and the recently identified type VI [33], [34], [35], [36], through which it secretes invasion antigens, such as CiaB, which may promote cellular invasion of the intestinal epithelial cells [37] . Furthermore, *C. jejuni* produces various toxins including CdtA-C, which have been reported to promote cellular cytotoxicity and apoptotic cell death [31].

More recently, whole genome investigation followed by *in vitro* analysis of the emerging gastrointestinal pathogen *C. concisus* revealed potential components contributing to the organism's pathogenesis; including several toxins, invasins in addition to colonisation, and adhesion factors [5], [31], [38], [39]. Studies by Man *et al.* [26] report that in addition to a transcellular route of invasion, *C. concisus* UNSWCD preferentially attaches to intercellular junctional spaces facilitating translocation across the epithelium, thus promoting a paracellular route of invasion [20], [40].

A likely reason for our current lack of knowledge regarding pathogenic mechanisms of *C. ureolyticus* is the lack of genomic data: until now the potential virulence apparatus of *C. ureolyticus* has remained unknown. Herein, we provide the first whole genome analysis of two *C. ureolyticus* strains. A comparative bioinformatics based approach was performed to identify putative virulence factors, secreted proteins and genomic heterogeneity of the two *C. ureolyticus* isolates in an investigation of the pathogenic mechanisms of this emerging pathogen.

## Materials and Methods

### Strains used in this study

The *C. ureolyticus* strains used in this study are outlined in Table 1.

**Table 1:** Strains used in this study.

Strain	Gender	Age (Years)	Sample Source	Medical Summary/Additional Information	Availability of whole genomic data
CIT001	Male	83	Faeces	Long stay psychiatry unit	In process (Our group)
CIT002	Female	84	Faeces	End stage chronic renal disease	In process (Our group)
CIT004	Female	3	Faeces	Admitted to hospital overnight with D/V and fever. Had *Cryptosporidium* oocysts	In process (Our group)
CIT005	Female	3	Faeces	D/V. Had *Cryptosporiium* oocysts	In process (Our group)
CIT007	Female	84	Faeces	End stage chronic renal disease	In process (Our group)
CIT009	Female	83	Faeces	Nursing home resident	In process (Our group)
DSM 20703	Female	Unknown	Amniotic Fluid	Type Strain; originally isolated in 1978	Assembled
ACS-301-Sch-V-3b	Female	Unknown	Vagina	A reference genome for the Broad Institute as part of the ‘The Human Microbiome Project’	Assembled

### Genome assembly

Sequence data for *Campylobacter ureolyticus* ACS-301-V-Sch3b were obtained from the Sequence Read archive (SRA), having been collected by the Broad institute as part of the Human Microbiome Project for use as a reference genome for the *Campylobacter ureolyticus* species, but which had yet to be assembled. The sample was isolated from the female vaginal tract and had been sequenced using Illumina HiSeq with 100 bp pair end reads. The accession number to the SRA raw data is SRX115248.

*C. ureolyticus* has not been previously sequenced so no reference genome is currently available for assembly. Additionally, *Campylobacter* strains of the same species have previously been shown to display large variation within the overall gene content of their genomes whereby distinct genomospecies have been identified, thought to be mainly attributable to horizontal gene transfer and gene loss. For strains with genomes divergent from their closest references or in the absence of a reference genome, reference-sequence guided assembly methods can provide limited genome definition; therefore a *de novo* assembly method was used.

The Velvet assembly tool [41] was used due to its compatibility with Illumina data and having been shown to be one of the best performing assembly programs available for paired end data [42]. Using Velvet a range of k-mer values from 39 to 59 (which determine the minimum read overlap) was tested to find the optimum hash length for assembly of the data. Velvet is based on a directed graph representation called de Bruijn graphs which uses non-redundant sets of k-mers or word length rather than sequence reads as its primary data structures. For the paired end reads a k-mer value of 53 was seen to be the optimal hash length. This information was subsequently used to carry out the assembly using Velvet, giving an N50 of 60,555 and a maximum contig length of 227,136 bp with a total of 115 contigs and a genome size of 1659961 bp, having removed contigs with a length of less than 100 bp.

(Note: The Accession Number to access *C. ureolyticus* DSM 20703 scaffolds is KB894730–KB894764 The location of the *C. ureolyticus* ACS-301-V-Sch3b scaffolds is: https://olive.broadinstitute.org/genomes/camp_ureo_acs-301-v-sch3b.1)

### Degree of diversity within *Campylobacter ureolyticus*

In addition to using the comparative genomics modules in RAST [43] and IMG/ER [44], we also conducted customized homology searches using BLAST (blastp, tblastn) and STRING to determine probable orthologs of genes conserved between the two *C. ureolyticus* strains and other species within the *Campylobacter* genus.

Bidirectional homology searches were performed on the two *C. ureolyticus* strains whereby cut off values set at 25% amino acid identity plus a minimum of 85% coverage were employed to identify the percentage of unique protein between both *C. ureolyticus* strains. A cut off a maximum 25% identity was employed to exclude homologues. The results obtained were compared with that of searches between the validated genomes of 4 *C. jejuni* strains within the RAST database; *C. jejuni* NCTC 11168, *C. jejuni* RM1221, *C. jejuni* 260.94 and *C. jejuni* 81-176. Cut-off values of 70% identity with a minimum of 85% gene length coverage were employed. A cut off value of a minimum of 70% identity was employed to identify highly conserved genes. It is important to note that in this study we considered protein sequences with >25% identity to be homologous, however those with >70% identity we conserved them to be highly conserved. Using the same parameters with *C. jejuni* NCTC 11168 set as the reference genome and 3 *C. jejuni* strains RM1221, 260.94 and 81-176 were individually and jointly compared to determine degree of identity at the amino acid level.

Furthermore, to identify the protein coding genes that are conserved between *Campylobacter* species, the genome of both *C. ureolyticus* strains DSM 20703 and ACS-301-Sch-V-3b were each set as reference genomes and compared to the protein coding genes within the genomes of all the available *Campylobacter* species in the RAST database; *C. jejuni* NCTC 11168, *C. jejuni* subsp. *doylei* strain 269.97, C. coli RM2228 [B], *C. lari* RM2100, *C. upsaliensis* RM3195, *C. fetus* subsp. *fetus* 82-40, *C. curvus* 525.92, *C. concisus* 13826 and *C. hominis* ATCC BAA-381. The minimum cut off limit for highly conserved genes was set to 70% amino acid identity using the RAST sequence based comparison tool. To identify genes unique to *C. ureolyticus* (ie: no homologues in the other *Campylobacter* species analysed) the maximum cut off identity value was set to 25% for the *Campylobacter* species mentioned above, additionally *C. ureolyticus* ACS-301-Sch-3b was included as a comparison genome where by the cut off identity value was set at a minimum of 70%. This allows for the identification of proteins conserved across the two *C. ureolyticus* strains but absent in other *Campylobacter* species.

### Gene prediction, identifying orthologs and synthenic associations

*C. ureolyticus* strains DSMZ 20703 (type strain) and ACS-301-V-Sch3b isolated from the female vaginal tract are the first *Campylobacter ureolyticus* strains to have been sequenced.

The *C. ureolyticus* DSMZ 20703 genome (IMG submission ID 11117, NCBI project ID 174981) and fasta sequences for 1799 protein coding genes were downloaded from the IMG/ER website. The RAST [43] web application server was used for gene predictions using the Glimmer3 [45] program.

Furthermore, functional domain analysis was conducted with Pfam and the Kyoto Encyclopedia of Genes and Genomes, available at (http://www.genome. jp/kegg) [46], was employed to determine the biochemical pathways to which genes were assigned.

The Search Tool for the Retrieval of Interacting Proteins (STRING), a database of known and predicted protein–protein interactions available at http://string.embl.de/, was employed to examine functional relationship between proteins across multiple species [47].

BlastP (NCBI database) and Atlas T4SS (http://www.t4ss.lncc.br/) were employed to identify similarity between the type IV secretion systems within in *C. ureolyticus* ACS-301-V-Sch3b to other organisms. Atlas T4SS is a database describing a large number of proteins related to the type IV secretion system reported in both Gram-negative and Gram-positive bacteria, as well as in Archaea.

### Comparative bioinformatic analyses

Homology searches were performed using the blastp and blastn tools through the National Centre for Biotechnology Information (NCBI) website (available at http://www.ncbi.nlm.nih.gov/) using the default settings. Comparative bioinformatic analyses on the genomes of *C. ureolyticus* strains DSM 20703 and ACS-301-V-Sch3b were performed using the RAST database. Both functional and sequence based analysis were performed using default settings.

### Secretome Prediction

The presence and location of signal peptide cleavage sites in the amino acid sequences were predicted using the default settings for Gram-negative bacteria on the SignalP Server 4.1 [48] (http://www.cbs.dtu.dk/services/SignalP/). Non-classically secreted proteins were predicted using the SecretomeP 2.0 Server [49] (http://www.cbs.dtu.dk/services/ SecretomeP/). SecretomeP predicts the possible secretion of proteins following signal peptide independent secretion pathways.

The statistical cut off was the default setting for both SignalP4.1 and the SecretomeP2.0 servers.

As a comparison, using identical parameters set for the *C. ureolyticus* analysis, the total percentage of *C. jejuni* NCTC 11168 secreted proteins were predicted using both the SignalP4.1 and SecretomeP2.0 servers. The amino acid sequences of *C. jejuni* NCTC 11168 protein coding genes were extracted from the IMG/ER annotation pipeliner server.

### Culturing of bacterial strains

*C. ureolyticus* strains were inoculated on to blood agar plates (Columbia Blood Agar base; Sigma Aldrich, with 5–7% defibrinated horse blood; Thermoscientific) supplemented with 2.5 g/L of Sodium Formate (Sigma Aldrich), 2.5 g/L Sodium Fumarate (Sigma-Aldrich) and 20 µg/mL of vancomycin (Sigma-Aldrich) [6]. The strains were grown under anaerobic conditions (AnaeroGen Gas Generating Systems Oxoid) at 37°C for 48 hours. The identity of the cultures were confirmed by) the presence of flat, spreading colonies on the blood agar plates, positive urea slants (Christensen's Urea Agar, Sigma-Aldrich) and the presence of slender Gram-negative rods under a light microscopy.

### Validations of syntenic associations

Bacterial DNA was extracted using the QIAamp DNA Mini Kit (Qiagen, Manchester, UK) according to the manufacturer's instructions. The concentration and quality of DNA was measured using a Nanodrop ND-1000 Spectophotometer (Nanodrop Technologies, Fischer Scientific,Ireland). To conifirm the presence of *C. ureolyticus* DNA in the samples following DNA extraction, *C. ureolyticus* specific PCR targeting the *hsp60* gene was conducted as described by Bullman et al. [4]. The confirmation of the presence/absence of gene that were shown to be present in *C. ureolyticus DSM 20703* but absent in ACS-301-V-Sch3b by next-generation sequencing was performed on the DSM 20703 strain along with 6 *C. ureolyticus* clinical isolates using PCR.

Primer pairs were designed to amplify regions (2,861, 1,625, 1,469, 311 bp) within several ORF clusters found in either DSMZ 20703 or ACS-301-V-Sch3b (Table 2). All PCR amplifications were performed in a 50 µl reaction volume, containing 3 µl DNA template, 1 U HotStarTaq DNA Polymerase (QIAGEN, West Sussex, UK; 203205); 5 µl of 10× PCR buffer and 1 µl 25 mM MgCl_2_ (provided with HotStarTaq DNA Polymerase), 8 µl of dNTPs mixture (1.25 mM of each dNTP; Sigma-Aldrich Ireland Ltd. Arklow, Ireland), and 2 µl of each primer (25 pmol/µl; Eurofins MWG Operon, London, UK), 29 ul molecular grade water. The thermal cycling conditions for all 4 reactions were: 94°C for 5 min, 35 cycles of 94°C for 30 s, Annealing temperature as specified in Table 1 for 30 s, and 72°C for 1 min per kb to be amplified, followed by 72°C for 5 min. PCR products were electrophoresed through 1.5% agarose gels at 100 V for 40–60 min. The products were then purified using the QIAquick® PCR Purification Kit (Qiagen, Manchester, UK) according to manufacturer's instructions. PCR positive products were sequenced by MWG (Eurofins, Germany) and analysed by Clustal W [50].

**Table 2:** Primers used in this study.

Target	Primer	Sequence 5′ – 3′	Annealing (°C)	Product (bp)	Source
*rtx*	71RTX F	CCT TAG CTC TTT TAT CAA GCG ATG	58	2,861	This study
	2932RTX R	CAC TCT TAT CGA TTG TAA TAA AGC C			This study
*virD4*	262VirD4*F	GAA ATG CAA GAT TTG CAA ACT CAG C	57	1,625	This study
	1887VirD4*R	CTC AGC ATC TTC TTC TAT TGG C			This study
*ciaB*	266CiaB F	TAC ATG AAA ACT CTC ATA GAA ATT TAA TC	56	1,469	This study
	1735 CiaB R	AGT AGT AGA TAA CAA ACT CTT TTG CAT C			This study
***doc***	32Doc1 F	GTT TGC ATG ATG ATA TAA TGG ATG	54	311	This study
	343Doc1R	TGA GTT AAA TCA TCT TTT GTT ATC TC			This study

Expression of these genes under standard conditions (as described previously) was investigated by reverse transcriptase PCR (rt-PCR). Briefly total RNA was extracted by Roche high pure RNA extraction kit (Roche Diagnostics, Mannheim, Germany) as per the manufacturer's guidelines and the purity and concentration of the RNA was measured using a Nanodrop ND-1000 Spectophotometer (Nanodrop Technologies). The RNA extracted was normalised across all strains and cDNA was synthesised using Tetro cDNA synthesis kit (Bioline). Gene specific PCR was carried out on both RNA and cDNA to ensure absence of contaminating genomic DNA.

### SDS-PAGE

*Campylobacter ureolyticus* strains were grown on blood agar plates (as described previously), bacterial cells were harvested and washed twice in phosphate buffered saline (PBS). Soluble proteins were extracted from the packed cells using B-PER Protein Extraction Reagents (Thermo Scientific) as per manufacturer's guidelines. The B-PER Protein extraction reagent is a nonionic detergent that disrupts cells and solubilizes native proteins without denaturation. Soluble protein was quantified by the bicinchoninic acid method (Pierce) [51], using bovine serum albumin as the standard. Proteins were separated according to their electrophoretic mobility in a sodium dodecyl sulphate polyacrylamide gel (SDS-PAGE), as described by Laemmli *et al.*, [52]. Proteins were resolved on 12% (w/v) resolving and 5% (w/v) stacking acrylamide gels. Gels were stained using Coomassie Brilliant Blue G-250 (Bio-Rad). Phoretix 1D pro software (TotalLab Ltd.; Newcastle Upon Tyne, NE, UK) was employed to analyse the percentage similarities between the protein profiles of the two *C. ureolyticus* strains, and to cluster the lanes based on banding patterns.

## Results and Discussion

Confirmation of bacterial pathogenesis and virulence potential requires a multi-factorial approach encompassing epidemiological data, candidate gene identification and functional analysis. The recent deposition of two *C. ureolyticus* genome sequences (strains DSM 20703 and ACS-301-V-Sch3b) has for the first time facilitated the genetic analysis of the pathogenic potential of this species. The type strain DSM 20703, originally isolated in 1978 from human amniotic fluid [9], was sequenced as part of ‘The one thousand microbial genomes (KMG)’ project. Strain ACS-301-V-Sch3b was isolated from the human vaginal cavity and sequenced by the Broad Institute as a reference genome for ‘The Human Microbiome Project’.

### *Campylobacter ureolyticus* diversity

The complex taxonomy of typical and emerging *Campylobacter* species is well established [53], whereby certain isolates, although conforming to the phenotypic description of a particular species, may exhibit a large degree of heterogeneity at the genomic level [54], [55]. For instance the emerging pathogen *C. concisus* is composed of several genemospecies that are likely to have varying impacts on human health and disease [31], [39], [56].

In the current study, bidirectional homology searches between the type strain *DSMZ 20703* and the vaginal isolate ACS-301-V-Sch3b revealed that 75–79.5% of proteins were highly conserved (70% identity). Using the same parameters with *C. jejuni* NCTC 11168 set as the reference genome, individual and multiple comparisons to 3 other *C. jejuni* strains, revealed that 92% and 87% of proteins respectively are highly conserved. Such data suggest that substantial variation exists within these two *C. ureolyticus* genomes. Interestingly, average percentage identities for all homologs revealed that *C. ureolyticus* strains had a higher variation when compared to the phylogenetically related species *C. jejuni* (94 vs. 98%, respectively).

Futhermore, whole genome comparison of the protein coding genes of the two *C. ureolyticus* strains against other members of the *Campylobacter* genus revealed that 9–22% of proteins were highly conserved across different *Campylobacter* species (Table 3). The greatest number of highly conserved homologs were present in *C. hominis* followed by *C. consisus* then *C. curvus* and *C. fetus* subsp. *fetus*. There are approximately 128 protein coding genes that were determined to be highly conserved across all *Campylobacter* species test when using *C. ureolyticus* DSM 20703 as the reference genome. These proteins have functions ranging from stress response, membrane transport, respiration and the metabolism of macromolecules (Table S1).

**Table 3:** Comparison of unique and conserved protein coding genes of *C. ureolyticus* against other *Campylobacter* species.

	*C. ureolyticus* DSM 20703		*C. ureolyticus* ACS-301-sch-3b	
Comparison Species	Unique Proteins (%)	Highly Conserved Proteins (%)	Unique Proteins (%)	Highly Conserved Proteins (%)
*C. jejuni* NCTC 11168	684 (38)	183 (10)	614 (36)	181 (11)
*C. jejuni* subsp. *doylei* 269.97	662 (37)	184 (10)	583 (34)	183 (11)
*C. coli* RM2228 [B]	689 (38)	181 (10)	591 (35)	179 (10.5)
*C. lari* RM2100	716 (40)	184 (10)	619 (36)	187 (11)
*C. upsaliensis* RM3195	726 (40)	169 (9)	619 (36)	169 (10)
*C. fetus* subsp. *fetus* 82-40	600 (33)	271 (15)	523 (31)	276 (16)
*C. curvus* 525.92	546 (30)	268 (15)	487 (29)	268 (16)
*C. concisus* 13826	520 (29)	279 (15)	486 (29)	285 (17)
*C. hominis* ATCC BAA-381	508 (28)	371 (20)	405 (24)	377 (22)
Average value	628 (35)	232 (13)	574 (32)	234 (14)

On the other hand *C. upsaliensis* RM3195 and *C. lari* RM2100 shared the lowest percentage of highly conserved protein coding genes and up to 40% of *C. ureolyticus* proteins did not contain homologs within the genomes of these species (Table 3). A total of 65 proteins were highly conserved between the two *Campylobacter* species (>70% identity) yet had no homologs (<25% identity) in any of the other *Campylobacter* species tested (Table S2). The majority of these proteins (n = 45) were classed as hypothetical and miscellaneous (n = 9). Additionally, the urease operon was also presented within this group (Table S2); however it is important to note that *C. sputorum* biovar. *paraureolyticus* and subset of *C. lari* UPTC (urease positive thermophilic *Campylobacter*) also contain this operon [57], [58]. As the whole genomes of these species are currently unavailable, they were not included in the comparison analysis.

**Figure 1:**
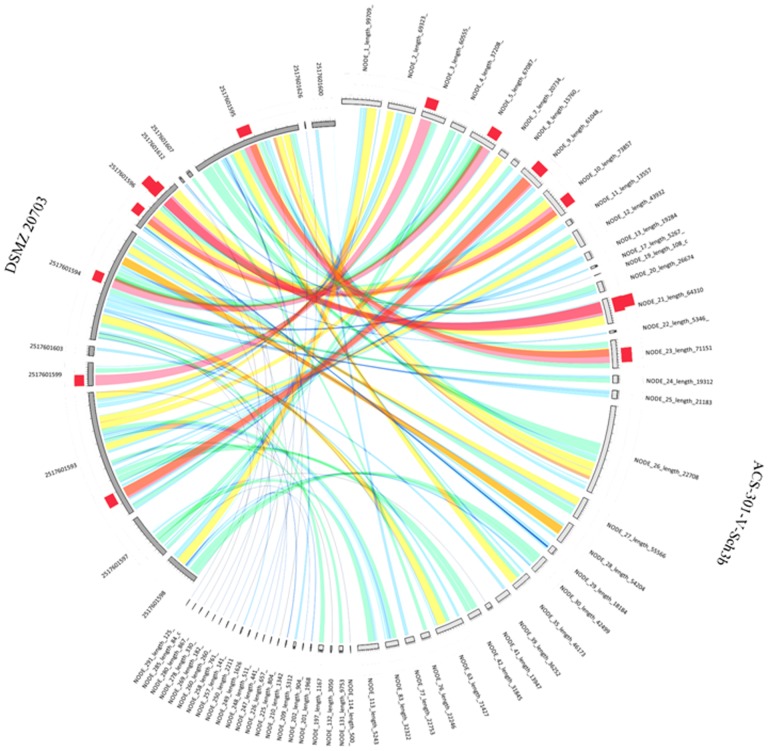
DSM 20703 V's ACS-301-V-Sch3b Synteny Map. Syntenic affinities (conserved blocks) across the whole genome sequences of ACS-301-V-Sch3b and DSM 20703 were determined using BLAST+ [133]with DSM 20703 used to build the database and using BLASTN to find synteny within the ACS-301-V-Sch3b genome. The ideogram was built using Circoletto [134] where ribbons represent the local alignments and the colours blue, green, orange and red, respectively representing the 25% blocks up to the maximum score of 100% and histogram on top of the ideograms, counting how many times each band has hit the specific part of the sequence.

While the genomes of *C. ureolyticus* DSMZ 20703 (1.74-Mb) and ACS-301-V-Sch3b (1.66-Mb) have a similar estimated size, analysis indicated that 18.8% (341/1810) and 17.1% (290/1700) of their protein coding genes are unique (Fig. 1, Table S3 and S4). Based upon studies by Goris and colleagues [59], the variation we observe between the *C. ureolyticus* strains suggests species delineation, whereby the cut off value for species is 85% conserved genes (15% unique protein coding genes) for a pair of strains. Such heterogeneity is likely to result in substantial functional differences between the two strains both from a general housekeeping and virulence perspective.

**Figure 2:**
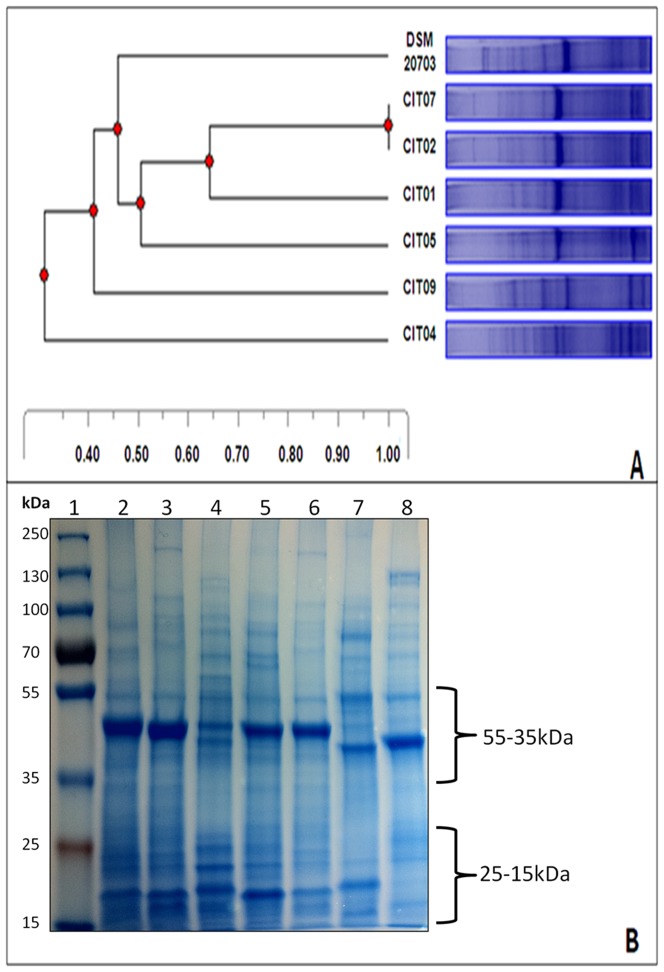
SDS-PAGE of *C.* *ureolyticus* Soluble Proteins. (A) Protein profiles of 7 *Campylobacter ureolyticus* strains. Phoretix 1D pro was employed to cluster the lanes based on banding patterns. The Dendrogram is an Unweighted Pair Group Method with Arithmetic Mean (UPGMA) Dice coefficient distance tree. (B) Original SDS-PAGE gel, with variable regions highlighted. Lane 1: Page Ruler Plus Ladder, lane 2: CIT001, lane 3: CIT002, lane 4: CIT004, lane 5: CIT 005, lane 6: CIT007, lane 7: CIT009, lane 8: DSM 20703.

In addition to the high degree of genomic variation observed between DSMZ 20703 and ACS-301-V-Sch3b strains, protein profiles of a further 6 *C. ureolyticus* isolates, whereby strains are clustered based on their banding patterns (Fig. 2), further confirms the significant degree of heterogeneity that exists between strains. A large scale whole genome analysis project including 12 C. *ureolyticus* strains, isolated from patients with diarrhoeal illness, asymptomatic patients and animal reservoirs is currently under way within our lab. Comparisons of whole genome coding sequences between these strains support our initial observations of substantial heterogeneity between *C. ureolyticus* strains. Paired genome comparison of the coding genes of 14 *C. ureolyticus* strains revealed that 2–20% of their proteins are unique. Furthermore, individual comparisons of the protein coding genes of the 12 *C. ureolyticus* isolates against *C. ureolyticus* DSM 20703 and *C. ureolyticus* ACS-301-Sch-V-3b revealed that 13–19% (average 16%) and 9–16% (average 12%) of proteins are unique respectively. Such heterogeneity between the *C. ureolyticus* type strain and isolates raises caution regarding its suitability as the type strain for this species.

Our preliminary data suggest that as with *C. concisus*, *C. ureolyticus* is likely composed of several genomospecies; however, additional large scale investigations will be required to determine the extent of genomic variations between strains of different origin and the consequences that such differences may have on their pathogenesis and virulence potential (this is the subject of continuing work in our lab).

### The *C. ureolyticus* secretome

A significant variant in contributing to bacterial pathogenic potential is the secretome – the totality of secreted proteins - characterised by its dynamic nature, undergoing variations and adjustments to match that required by the prevailing environmental conditions [60]. The secretome accounts for a significant proportion of the total bacterial proteome and is likely to contain a number of important virulence or virulence associated factors including colonization and stress survival factors [61]. As such, *in silico* based predictions of the *C. ureolyticus* secretome can assist in cataloguing the strain's pathogenic potential.

**Figure 3:**
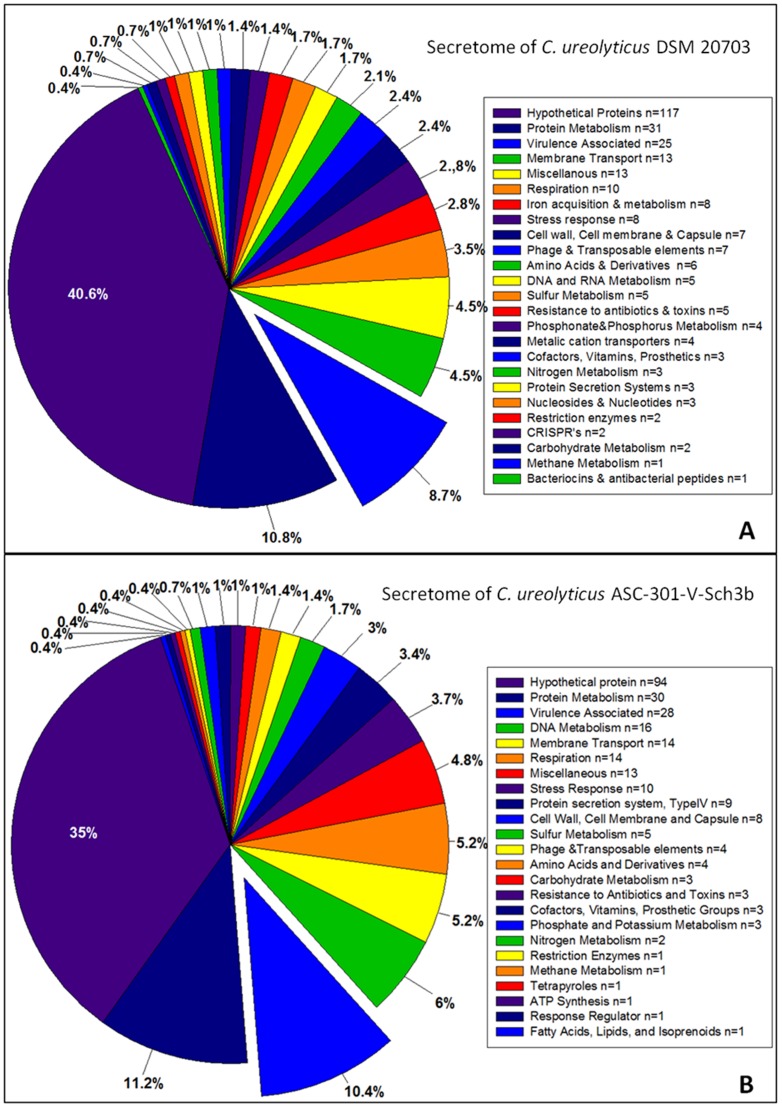
Bioinformatics based predictions of the *C.* *ureolyticus* secretome. (A) 288 proteins were predicted to be secreted by *C. ureolyticus* DSMZ 20703 using the SignalP 4.1 server for classically secreted proteins and the SecretomeP 2.0 server for non-classically secreted proteins. Of the 288 proteins, 187 proteins were predicted to be secreted by the SecretomeP 2.0 sever alone, 57 proteins by the SignalP 4.1 alone and 44 proteins were identified by both servers. Additionally, 117 proteins of 28 were hypothetical, the remaining 171 proteins were associated with a diverse range of functions relating to membrane transport, protein metabolism, respiration, stress response and at least 25 proteins with putative virulence roles. (B) SignalP4.1 and SecretomeP2.0 servers predicted 269 proteins are secreted by *C. ureolyticus* ACS-301-V-Sch3b. 165 proteins were predicted to be secreted by the SecretomeP 2.0 server alone, a further 65 proteins were identified by the Signal 4.1 server and 39 proteins were identified by both servers. Of the 269 secreted proteins, a total of 94 were hypothetical proteins, the remaining 175 proteins were predicted to have a wide range of functions including DNA metabolism, cell wall synthesis, sulphur and nitrogen metabolism and at least 28 proteins with putative virulence roles.

A total of 288 proteins were predicted to be secreted by *C. ureolyticus* DSMZ 20703 (Table S5) including at least 25 proteins with putative virulence roles (Fig. 3a). Additionally, the secretome of ACS-265 was predicted to contain 269 proteins (Table S6), 28 of which have proven roles in virulence (Fig. 3b).

Although these results are slightly lower than has been reported for *C. concisus* 13826 when using SignalP3.0 [38], our figures align closely to the predicted secretome of *C. jejuni* NCTC 11168. A combination of SignalP4.1 and SecretomeP2.0 analysis, predicted that a total of 256 proteins (15.5%) were secreted by either classical or non-classical pathways by *C. jejuni* NCTC 11168. SignalP4.1 predicted 115 proteins were classically secreted and SecretomeP predicted 200 proteins were secreted by a signal peptide independent manner, however 59 of these proteins overlapped between the two servers.

These putative secreted virulence factors are of particular interest in light of a recent report by Burgos-Portugal *et al* [21], which showed that the secretome of *C. ureolyticus* is toxic to host cells, significantly reducing cell viability in epithelial cell lines. It is important to note that these proteins are only predicted to be secreted and remain to be imperially proven.

### Genomic variations unique to *C. ureolyticus* DSMZ 20703; a virulence perspective

Given that less than 83% of proteins are homologs between the two *C. ureolyticus* strains (Table S7 and S8), we identified 341 genes unique to DSMZ 20703 when compared to ACS-301-V-Sch3b, encoding proteins with a variety of functions; including capsular and extracellular polysaccharide formation, iron acquisition, metabolism, transport systems, phage components, stress response, 16 putative virulence factors subcategorised into toxins and adhesions, as well as 242 hypothetical proteins (Table S4).

### Toxins

We identified 13 haemolytic cytotoxins and cytolysin related proteins, of which 8 were predicted to be secreted (Table 4). Owing to their ability to increase the availability of iron during the process of infection [62], such pore-forming toxins represent an important component of a pathogen's virulence repertoire. Indeed, cell-associated and secreted haemolysins/cytolysins in the well-studied *C. jejuni* and several of the emerging *Campylobacter* species have previously been noted as potential contributors to Campylobacter gastroenteritis [62], [63].

**Table 4:** Putative virulence associated genes in *C. ureolyticus* DSM 20703.

Gene Function	Accession No.	Homolog in ACS-301-V-Sch3b	Secreted
**Type IV pili (T4P) Formation**			
Twitching motility protein PilT	KC907219	Yes	No
Type II secretion envelope pseudopilin protein (PulG,guides folded protein to PulD in outer membrane)	KC907220	Yes	No
Type II secretion envelope pseudopilin protein (PulG,guides folded protein to PulD in outer membrane)	KC907221	Yes	No
Type IV pilus biogenesis protein PilQ	KC907222	Yes	No
Type IV pilus biogenesis protein PilQ	KC907223	Yes	No
Type IV pilus biogenesis protein PilQ	KC907224	Yes	Yes
Leader peptidase (Prepilin peptidase)/N-methyltransferase	KC907225	Yes	No
Type II secretory pathway, ATPase PulE/Tfp pilus assembly pathway, ATPase PilB	KC907226	Yes	No
putative prepilin-type N- cleavage/methylation domain protein	KC907227	Yes	Yes
Type II secretion envelope pseudopilin protein (PulG,guides folded protein to PulD in outer membrane)	KC907228	Yes	Yes
Twitching motility protein PilT	KC907229	Yes	No
**Factors involved in colonisation and adhesion**			
Autotransporter adhesin with YadA-like domain	KC907230	Yes	Yes
exoprotein with autotransporter precursor	KC907231	Yes	Yes
FlpA; Type III fibronectin domain-containing lipoprotein	KC907232	Yes	Yes
Fibronectin/fibrinogen-binding protein	KC907233	Yes	No
CadF; Outer membrane fibronectin-binding protein	KC907234	Yes	Yes
Death-on-curing family protein	KC907235	Yes	No
Death-on-curing family protein	KC907236	Yes	No
(HecA) Filamentous Hematagluttinin/Putative large exoprotein involved in heme utilization or adhesion	KC907237	No	No
(HecA) homolog Filamentous Hematagluttinin/Putative large exoprotein , heme utilization or adhesion	KC907238	No	No
Two-component system response regulator RacR	KC907239	Yes	No
Major outer membrane protein (Cmp/PorA)	KC907240	Yes	Yes
PAL; Peptidoglycan associated lipoprotein Omp 18	KC907241	Yes	Yes
CjaC homolog	KC907242	Yes	Yes
Amino acid ABC Transporter/PEB1 (surface antigen) homolog	KC907243	No	Yes
Outer membrane lipoprotein omp18 precursor/CjaD	KC907244	Yes	Yes
Capsule biosynthesis protein capA	KC907245	Yes	No
CjaA surface adhesion	KC907246	Yes	Yes
PEB4; Major antigenic peptide	KC907247	Yes	Yes
**Hemolysins**			
Putative hemolysin	KC907248	Yes	No
Probable hemagglutinin/S-type Pyocin/Colicin E3 (cytotoxic domain)	KC907249	No	Yes
TlyC; Hemolysins and related proteins containing CBS domains	KC907250	Yes	No
TlyC; Hemolysins and related proteins containing CBS domains	KC907251	Yes	No
Channel-forming transporter/hemolysin activation protein (HecB) TVSS	KC907252	No	No
Filamentous haemagglutinin family; VacA-like (serine protease)	KC907253	No	Yes
**RTX related cytotoxins**			
RTX haemagglutinin, iron-regulated protein FrpC (N. meningitidis) related to hly	KC907254	No	Yes
RTX haemagglutinin, iron-regulated protein FrpC (N. Meninfitidis) related to hly	KC907255	No	Yes
RTX-Hemolysin-type calcium-binding region:FrpC related	KC907256	No	Yes
RTX related Ca binding hemolysin (FrpC homolog)outer membrane adhesin-like protein, partial	KC907257	No	Yes
Calcium binding hemolysin protein (Not in other Campy spp)/LeukotoxinA (lktA)/hlyA	KC907258	No	No
Haemolysin type calcium binding protein RTX related	KC907259	No	Yes
**Toxins**			
Vacuolating cytotoxin paralog VacA	KC907260	Yes	Yes
Addiction module toxin, RelE/StbE family	KC907261	Yes	No
Zona occludens toxin	KC907262	Yes	No
Zona occludens toxin	KC907263	Yes	No
S-layer RTX protein	KC907264	No	Yes
**Invasion**			
Campylobacter invasion antigen B (CiaB)	KC907265	Yes	No
PldA; Phospholipase A1 precursor; Outer membrane phospholipase A	KC907266	Yes	Yes
U32 Pepsidase; collagenase family	KC907267	Yes	No
Cell wall-associated hydrolases (invasion-associated proteins)	KC907268	Yes	Yes
**T6SS**			
IcmF-related protein	KC907269	No	No
**Type II secretion system**			
MSHA biogenesis protein MshL	KC907270	Yes	Yes
MSHA biogenesis protein MshM	KC907271	Yes	No
**Type I Secretion system**			
Type I secretion membrane fusion protein, HlyD/LktD (Hemolysin secretion protein)	KC907272	No	No
Hemolysin secretion protein HlyB/Leukotoxin translocation; LktB	KC907273	No	No

Within this group, at least 8 repeats-in-toxins (RTX) related proteins, 6 of which were predicted to be secreted were identified. Various numbers of such repeats are found in the RTX domains of several cytotoxins/leukotoxins involved in the virulence of a number of different Gram-negative genera [64]. A characteristic feature of these exoproteins is the use of type I secretion systems to facilitate protein export across the bacterial envelope into the extracellular space [65]; their functionality being limited by Ca^2+^ ion availability, which serves to sequester activity until outside of the bacterial cell [65].

Furthermore, 3 of these secreted RTX haemolysins appear to be iron-regulated, with homology (up to 52% identity over the entire amino acid sequence) to the FrpC RTX protein of pathogen *Neisseria Meningitisis*. Intriguingly, Osička *et al* [64], reported the detection of FrpC-specific antibodies in the sera of patients recovering from invasive meningococcal disease, demonstrating that the FrpC-like protein is produced *in vivo* during infection, though its exact role in the infectious process remains unknown [66], [67]. Akin to the emerging pathogen *C. concisus*, the presence of iron-regulated haemolysins in *C. ureolyticus* suggests a potential function for such haemolysins as an important component in promoting human disease [62].

Additionally, a secreted S-layer RTX protein exhibiting 79% identity over the entire amino acid sequence of that of *C. concisus* was detected. Similarly, a study by Burgos-Portugal [68], which focused on the secreted proteins of *C. ureolyticus* UNSWCD, isolated from an intestinal biopsy of a child with Crohn's disease, identified an S-layer RTX protein. Such S-layer proteins are thought to render resistance to complement while also providing structures for adherence of the pathogen to the host cell [69]. S-layer RTX is a pore-forming toxin that is also found in *Campylobacter rectus* and toxins within this family are recognized as true virulence factors [65], [70].

### Adhesins

Bacterial adhesions are important in establishing initial infection, permitting host cell interaction and are often a prelude to later pathological events [71]. Within the group of haemolysins unique to DSM 20703, two genes encoding the HecA protein, a member of the filamentous hemagglutinin (FHA) family, were detected. Furthermore, directly upstream of one of the *hecA* genes, the *hecB* locus, coding for a haemolysin activation protein was also identified. The HecA/B proteins make up a two-partner secretion (TPS) system, whereby a TpsA family exoprotein containing a specific conserved secretion signal is recognised by a TpsB family channel-forming transporter allowing it to cross the membrane [72].Rojas and colleagues [73] report that *HecA* homologs are found in both animal and plant pathogens and interestingly within animals appear to be restricted only to pathogenic species. Furthermore, HecA has been identified as an adhesion contributing to the virulence of *C. jejuni* [74].

Interestingly, the 5.8 kb *hecA* gene within the HecA/B operon has a G+C content 17% lower than the rest of the *C. ureolyticus* genome (G+C content 29%). Given that lateral gene transfer of *hecA* genes has previously been noted in other species [75], it is not unreasonable to suggest that this adhesion may have been acquired from a pathogen outside the Campylobacter genus. One possible source of *hecA* (based on G+C content and signature codon usage) is the *Fusobacterium* species; which, similar to *C. ureolyticus* [4], [19], [76], have been linked to periodontitis and gastrointestinal disease including irritable bowel disease (IBD) and intermittent colitis [77], [78]. It is likely that such genetic transfer may have occurred when these bacteria occupied the same ecological niche [79].

Two further adhesions identified in the current study are the PEB1 homolog and the intracellular multiplication factor protein IcmF [80], [81]. PEB1 is a known colonization and virulence factor in *C. jejuni*; with Pei *et al* [82], reporting that a mutation in the PEB1 resulted in 50- to 100-fold less adherence to, and 15-fold less invasion of, epithelial cells in culture. On the other hand, the IcmF protein, a component of a type VI secretion system (T6SS), has only recently been identified as playing a role in controlling bacterial virulence in eukaryotic host cells, along with mediating competition between bacteria [22], [33]. Lertpiriyapong *et al* [33], reported that *C. jejuni* use T6SS to establish persistent colonization. In addition to *IcmF*, we have identified homologs of several other T6SS genes in *C. ureolyticus*, including 2 *ompA/motB* genes, inner membrane proteins responsible for T6SS stabilisation [33]. Given that T6SS-associated genes appear to be present in emerging *Campylobacter* spp. [22], it may be valuable to investigate the acquisition and potential role of this secretion system in the pathogenesis of these emerging species.

Finally, a 30 kb region of the DSM 20703 genome contains genes involved in N-linked gylcosolyation including *pglG*, *pglA*, *pglB* and *pglI*, genes coding for flippase enzymes along with those implicated in exopolysacchaaride and lipopolysaccharide biosynthesis and modification (Table S2). Strikingly within this region, we have identified genes involved in Sialic Acid (N-acetylneuraminic Acid) metabolism. Of particular interest is a cluster of genes required for de novo synthesis of Sialic Acid; *siaC* (NeuB superfamily), *siaB* (CMP-Neu5Ac-synthase) and *siaA* (NeuC superfamily). Furthermore, analysis indicates these genes are homologous to those of *N. meningitidis* with 96–100% coverage and 50–76% identity over the entire amino acid sequences. Such genes are pivotal virulence factors to *N. meningitidis*, by studding its capsular polysaccharide and LOS with sialic acid it facilitates their ability to colonize, persist, evade host immune response and cause disease in mammalian species [83], [84].

Similarily, *C. jejuni* has three sets of *neu* genes involved in sialic acid biosynthesis, whereby it's thought a complete cluster (*neuB1, C1, A1*) has a role in LOS sialylation [85]. Sialic acid is an uncommon component of bacterial surface structures and, through molecular mimicry, may be crucial for evasion of host immunity and in post-infection autoimmune diseases such as Guillain–Barré syndrome [85], [86].

### Genomic variations unique to *C. ureolyticus* ACS-301-V-Sch3b; a virulence perspective

We identified 290 genes unique to ACS-301-V-Sch3b (Table S3), comprising proteins involved in cell wall and capsule biosynthesis, membrane transport, phage replication, stress response, DNA and protein metabolism, 20 putative virulence factors subcategorised into type IV secretion and toxin-antitoxin systems as well as 193 hypothetical proteins.

### Type IV Secretion systems (T4SS)

We identified 16 genes, 8 of which are predicted to be secreted, which appear to be involved in the archetypal VirB/D4 Type IV secretion apparatus (Table 5). Type IV secretion systems (T4SS) can be regarded as multi-subunit, molecular syringes, spanning the cell envelope to inject their specific substrate into the cytosol of target cells [87]. They are involved in conjugative DNA transfer in prokaryotes [88] and are exploited by several mammalian pathogens [89] for toxin secretion and targeted delivery of virulence factors into eukaryotic host cells during infection [90], contributing directly to pathogenicity [91].

**Table 5:** Putative virulence associated genes in *C. ureolyticus* ACS-301-V-Sch.

Gene Function	Accession No.	Homolog in DSM 20703	Secreted
**Type IV secretion system (T4SS)**			
Minor pilin of type IV secretion complex (VirB5)	KC465892	No	Yes
Inner membrane protein forms channel for type IV secretion of T-DNA complex, VirB4	KC465888	No	No
ATPase provides energy for assembly of type IV secretion complex& secretion of T-DNA complex (VirB11)	KC465883	No	No
Type IV secretion/competence protein (VirB10)	KC465893	No	Yes
Type IV secretion/competence protein (VirB9)	KC465894	No	Yes
VirB8	KC465897	No	No
VirB4	KC465896	No	No
Bores hole in peptidoglycan layer allowing type IV secretion complex assembly to occur (VirB1)	KC465885	No	Yes
Type IV secretion system protein VirD4	KC465895	No	No
Integral inner membrane protein of type IV secretion complex (VirB6)	KC465891	No	Yes
Inner membrane protein forms channel for type IV secretion of T-DNA complex, VirB8	KC465890	No	No
Forms the bulk of type IV secretion complex that spans outer membrane and periplasm (VirB9)	KC465887	No	No
Inner membrane protein forms channel for type IV secretion of T-DNA complex (VirB10)	KC465889	No	Yes
ATPase required for both assembly of type IV secretion complex and secretion of T-DNA complex, VirB11	KC465884	No	No
Coupling protein VirD4, ATPase required for T-DNA transfer	KC465886	No	No
IncQ plasmid conjugative transfer DNA nicking endonuclease TraR (pTi VirD2 homolog)	KC907204	No	Yes
Type IV sectreion/conjugative transfer protein TrbC (VirB2; pilins)	KC907205	No	Yes
**Adhesion and colonisation**			
Death-on-curing family protein	KC465877	Yes	No
Death on curing protein, Doc toxin	KC465876	Yes	No
Cell filamentation-like protein (Fic domain)	KC465874	No	No
Cell Surface Protein (YadA Domain protein)	KC465875	Yes	Yes
CadF; Outer membrane fibronectin-binding protein	KC465879	Yes	Yes
Fibronectin/fibrinogen-binding protein	KC465878	Yes	No
FlpA; Type III fibronectin domain-containing lipoprotein (String)	KC465881	Yes	Yes
Two-component system response regulator RacR	KC465882	Yes	No
Outer membrane lipoprotein omp18 precursor/CjaD	KC907206	Yes	Yes
Capsule biosynthesis protein capA	KC465850	Yes	No
CjaA; surface antigen	KC907207	Yes	Yes
CjaC	KC907208	Yes	Yes
PEB4; Major antigenic peptide	KC907209	Yes	Yes
Major outer membrane protein (Cmp/PorA)	KC907210	Yes	Yes
PAL; Peptidoglycan associated lipoprotein Omp 18	KC907211	Yes	Yes
**Invasion and intracellular resistance**			
Campylobacter invasion antigen B (CiaB)	KC465873	Yes	No
Cell wall-associated hydrolases (invasion-associated proteins)	KC907212	Yes	Yes
PldA; Phospholipase A1 precursor; Outer membrane phospholipase A	KC465880	Yes	Yes
U32 pepsidase; collagenase family	KC465849	Yes	No
**Toxins**			
Putative vacuolating cytotoxin (VacA) paralog	KC465870	Yes	Yes
Zona occludens toxin	KC465872	Yes	No
Zeta toxin	KC465871	No	Yes
Hypothetical protein; addiction module RelE/StbEtoxin	KC465869	Yes	No
MSHA biogenesis protein MshL pilus (Type ii and iii secretion system)	KC907213	Yes	Yes
MSHA biogenesis protein MshM	KC907214	Yes	No
**Hemolysins and Haemagluttinins**			
Putative hemolysin	KC465868	Yes	No
Hemolysins and related proteins containing CBS domains (TlyC)	KC465866	Yes	No
Hemolysins and related proteins containing CBS domains (TlyC)	KC465867	Yes	No
Hemagluttinin/autotransporter	KC907215	No	Yes
Hemagluttinin/autotransporter	KC907216	Yes	Yes
**Type IV pili**			
Type II secretory pathway, ATPase PulE/Tfp pilus assembly pathway, ATPase PilB	KC465857	Yes	No
Type IV pilus biogenesis protein PilQ	KC465858	Yes	No
Leader peptidase (Prepilin peptidase)/N-methyltransferase	KC465851	Yes	No
Putative prepilin-type N- cleavage/methylation domain protein	KC465854	Yes	Yes
Twitching motility protein PilT	KC465855	Yes	No
Twitching motility protein PilT	KC465856	Yes	No
Type II secretion envelope pseudopilin protein (PulG)	KC907217	Yes	No
Type II secretion envelope pseudopilin protein (PulG)	KC907218	Yes	No

The 16 putative *C. ureolyticus virB/D4* genes, appear to contain all of the necessary components for a structurally functional T4SS. Additionally, it is worth noting that *C. ureolyticus* has two copies each of the *virB* genes: *virB8*, *virB9*, *virB10* and *virB11*. A total of 13/16 and 10/16 of the VirB/D4 proteins in DSM 20703 were homologs to the *virB/D4* genes of *C. jejuni* and *C. fetus* subspecies *venerealis* with an average Identity of 55% and 60% respectively. Kienesberger *et al* [41], reported that mutational inactivation of the *virD4* and *virB9* components in virulent *C. fetus subsp. venerealis* isolates resulted in attenuated invasion and cell-killing phenotypes in cultured human cell lines, concluding that the VirB/VirD4 T4SS is necessary for efficient invasion and cytolethal damage [90], [92]. Additionally, *C. ureolyticus*, like a number of other pathogens [92], contains a FIC (filamentation induced by cyclic AMP) domain-containing protein downstream of the *virB-virD4* genes. FIC proteins disrupt host cell processes through AMPylation reactions on target proteins [93], and in *C. fetus* have been shown to be potential effector proteins translocated by the T4 machinery to mammalian cell [90], where they regulate host processes important to pathogen survival and replication [90].

Furthermore, the VirB/D4 genes in pathogen *C. jejuni*, located on the pVir plasmid, are likely to play an important role in bacterial invasion [94], [95], [96]. In support of this proposal, mutation analysis of the *C. jejuni virB11 gene*, carried out by Bacon *et al* [94], resulted in a 6-fold reduction in adherence and an 11-fold reduction in invasion leading to reduced virulence in a ferret model of infection. Subsequent studies from the same group reported that modifications to the *virB9* gene resulted in a significant reduction in *C. jejuni* invasion [95].

Additionally, it is worth noting that the ACS-301-V-Sch3b strain also contains 3 *tra* genes; *traL*, *traF* and *traJ* in addition to the *trb* operon, composed of at least 10 *trb* genes; *trbB*, *trbC*, *trbD*, *trbE*, *trbM*, *trbJ*, *trbL*,*trbF*, *trbG* and *trbI*. The products of these genes form part of a F-T4SS [97], however unlike the VirB/D4 genes associated with the P-T4SS, F-T4SS are most likely concerned with bacterial conjugation and DNA exchange [91].

### Toxin-Antitoxin system

Within the unique proteins of the ACS-301-V-Sch3b strain we detected a Zeta toxin. This kinase is usually co-expressed as part of a toxin–antitoxin (TA) module consisting of labile antitoxin (Epsilon) and a stable toxin (Zeta) in several pathogenic bacteria [98]. As Zeta toxins may provoke an autolytic phenotype, Meinhart and colleagues [98], speculate that the suicide of a few bacteria in a rapidly growing population may promote the release of other toxins that can attack their host cells or competing bacteria, thereby protecting their own. On the other hand, Lioy and colleagues [99], demonstrate that the Zeta toxin initially induces a set of protective responses, with selective up and down-regulation of particular genes to promote entry into dormancy rather than showing bactericidal behaviour. As the ability of TA systems to induce cell lysis or cell stasis has also been linked to biofilm and persister cell formation in pathogens [98], it would be interesting to investigate the prevalence of the Zeta toxin amongst other *C. ureolyticus* strains [98], [99], [100].

### Virulence determinants conserved between *C. ureolyticus* DSMZ 20703 and ACS-301-V-Sch3b

Amongst the 1,469 homologous proteins (Tables S5 and S6) shared between these two strains include a minimum of 35 putative virulence factors (Table 4 and 5) associated with motility and biofilm formation, adhesion and invasion.

### Type IV pili (TFP): twitching motility

Bacterial motility is an important factor in survival and pathogenesis [101]; however unlike most other *Campylobacter* species *C. ureolyticus* employs a flagellum-independent motility with the aid of type IV pili (TFP) [102], [103], [104]. At least 10 proteins associated with the formation of TFP were identified in both strains (Table 4 and 5). TFP are thin, flexible fibres displayed by a wide variety of Gram-negative bacteria whereby they use a modified version of the type II secretion system for their biogenesis. Such bacteria may employ TFP as linear actuators to enable directional crawling known as “twitching” motility, bearing resemblance to a grappling hook [6], [105], [106], [107], [108]. To negotiate significantly long distances (several microns) and orientate direction [100], multiple type-IV pili undergo cycles of repeated extension-adhesion and retraction-release using a “tug-of-war” mechanism [108], [109] driven by an ATP motor [110]. TFPs are known bacterial virulence factors supporting adhesion to host cells and abiotic surfaces, biofilm formation, motility, and horizontal gene transfer [105], [111], [112].

### Adhesion and Invasion

Amongst common features of many pathogenic microorganisms is their ability to utilize host cell factors to facilitate attachment and invasion. Of particular interest in the *C. ureolyticus* genome are 3 fibronectin associated proteins, 2 of which, *Campylobacter* adhesion to fibronectin (CadF) and Fibronectin-like protein A (FlpA), are predicted to be secreted. Fibronectin, a large glycoprotein, is a component of the extracellular matrix (ECM) of the human intestinal epithelium, serving as an adhesion molecule for many bacteria pathogens [113]. The CadF and FlpA proteins are reported as major virulence factors in *C. jejuni* and facilitate adhesion and colonization to host epithelial cells [113], [114]. Interestingly, a functional analysis of the *C. ureolyticus* secretome led Burgos-Portugal *et al* [21], to report the presence of a ‘CadF homologue’ which they suggest to be a significant contributing factor to the pathogenic potential of *C. ureolyticus* UNSWCD, presumably allowing for adhesion to and subsequent colonization of host cells.

Furthermore, CadF and FlpA have also been reported to be involved in the activation of the small Rho GTPases Rac1 and Cdc42 (by an as yet uncharacterised mechanism), enabling host cell entry [30], [115]. The fibronectin host cell-surface receptor is the α5β1 integrin which is located on the luminal surface of M cells in the gastrointestinal tract and may promote preferential binding [116]. However, in intact epithelia, this is restricted to the basolateral surface and as such is not readily available for interaction with luminally positioned microbial pathogens [117], [118]. Monteville *et al.* [119] demonstrated that adherence and internalization of *C. jejuni* were significantly increased by exposure of cellular basolateral surfaces, and that Fn was the receptor; suggesting that *C. jejuni* invasion may preferentially occur via a paracellular rather than an intracellular route.

**Figure 4:**
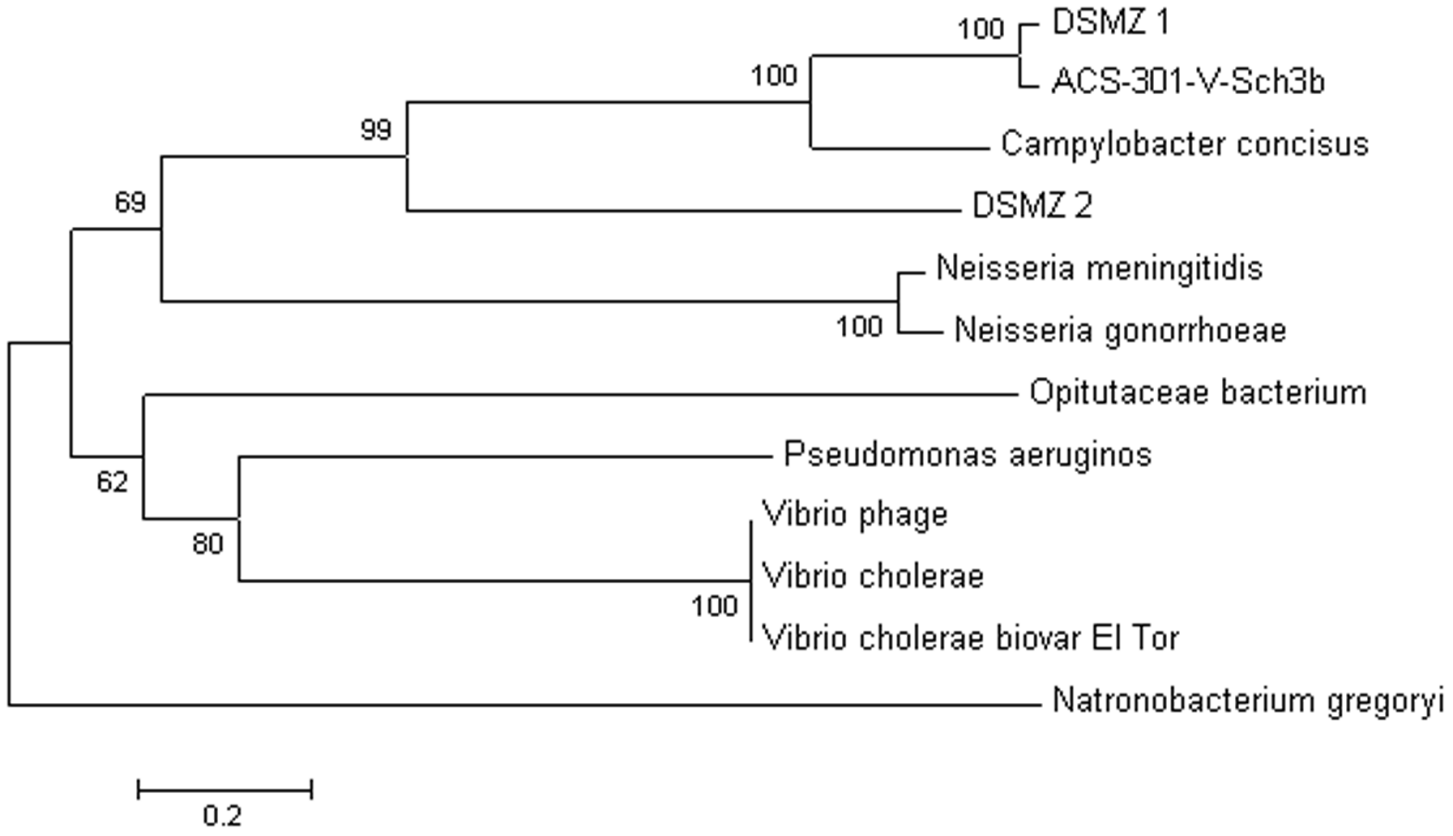
Evolutionary relationships of taxa Zona Occluden Toxin (ZOT). The evolutionary history was inferred using the Neighbor-Joining method. The percentage of replicate trees in which the associated taxa clustered together in the bootstrap test (1000 replicates) is shown next to the branches. The tree is drawn to scale, with branch lengths in the same units as those of the evolutionary distances used to infer the phylogenetic tree. DSMZ 1 and DSMZ 2 correspond to the ZOT paralogs in *C. ureolyticus* DSM20703. ACS-301-V-Sch-3b:*C. ureolyticus* ACS-301-V-Sch-3b.

Intriguingly, *in vitro* studies of *C. ureolyticus* UNSWCD indicate that this organism is capable of translocating across the cell monolayer, proposing that, as with *C. jejuni*, *C. ureolyticus* might also invade via a paracellular route [21]. In support of this hypothesis, in addition to the fibronectin binding proteins, we have also identified the zona occludins toxin (Zot) in both *C. ureolyticus* strains. The apical domain of epithelial cells is separated from the lateral domain by the zonula occludens, which forms the tight junction [120]. Zot is known to mimic a physiological modulator of intercellular tight junctions, and is used by virulent pathogens such as *Vibrio cholerae* and *Neisseria meningitidis* to induce a reversible opening of tight junctions between cells and increase the paracellular permeability in a non-toxic manner [121], [122]. Analysis indicates that Zot of *C. ureolyticus* bears greatest resemblance to that of *C. concisus* (Fig. 4), forming paraphyletic groups and thus are likely to have shared a common ancestor. As proposed for the emerging pathogen *C. concius* [5] and in agreement with *in vitro* studies [21], it is likely that *C. ureolyticus* is capable of attaching to and invading the host paracellularly. We suggest *C. ureolyticus* likely targets the host cell tight junctions, by expressing Zot, and binds to the basolateral surface of the cell via fibronectin binding proteins such as the secreted CadF and FlpA.

Additional factors which may promote *C. ureolyticus* adhesion, colonisation and invasion include the 4 secreted proteins CjaA, CjaC, PEB4 and Pal/Omp18. The CjaA, CjaC and Pal/Omp18 represent a surface-exposed protein, an ABC-transporter protein and a cell membrane-associated protein respectively. All 3 proteins are known to be highly immunogenic during human infection with the foodborne pathogen *C. jejuni* [123]. Moreover, the PEB4 protein is an antigenic virulence factor implicated in host cell adhesion, invasion, and colonization in *C. jejuni* [100].

A further two proteins, the Campylobacter invasive antigen (CiaB) and the secreted phospholipase A (PldA), were detected and are likely to contribute to the pathogenic potential of *C. ureolyticus*. PCR confirmed that the *ciaB* gene is common among *C. ureolyticus* strains (Fig. S1) and ClustalW alignment revealed that the nucleotide sequence of this gene is highly conserved between strains. Both CiaB and PldA have been linked to an invasive phenotype in *C. jejuni* [124]. Indeed, disruption of the *C. jejuni ciaB* gene, which encodes a protein that is translocated into the cytoplasm of eukaryotic cells, results in a non-invasive phenotype [125].

### Autotransporters

A protein family of particular interest, predicted to be present in both *C. ureolyticus* strains, are the autotransporters. These proteins represent an extensive and rapidly growing family contributing to bacterial virulence in Gram-negative bacteria such as *C. jejuni* and *H. pylori* [126], [127]. Although autotransporters share a common mode of secretion (similar translocation units), the passenger domains at the N-termal of the protein are highly diverse [128]. Almost without exception, all characterized passenger protein domains of autotransporters have been implicated in bacterial virulence relating to bacterial motility, adhesion, host immunomodulation, toxigenicity and intracellular spread [127], [128].

Although we have identified several autotransporter related genes (Table 4 and 5), the products of which have all been predicted to be secreted, of particular interest were the adhesion related autotransporters (2 copies of which are found in ACS-301-V-Sch3b) containing a YadA domain. The YadA protein is a major adhesin of *Yersinia pseudotuberculosis* which is noted to promote tight adhesion to mammalian cells by binding to extracellular matrix proteins [108]. Eitel *et al.* [108] reports the expression of YadA promotes both bacterial adhesion and high-efficiency invasion. Moreover, the YadA protein has been detailed to mediate the initial adhesion, uptake, and transfer of the bacteria through M cells of the intestine in addition to establishing extracellular colonization of the liver, spleen and underlying lymphatic tissue [108].

### Toxin-Antitoxin systems

Interestingly, two bacterial toxin–antitoxin (TA) systems RelE/StbE and the death on-curing (Doc) toxin were conserved in both *C. ureolyticus* strains. TA modules are highly abundant in opportunistic pathogens such as *Mycobacterium tuberculosis*, as mentioned previously when discussing the Zeta toxin, the ability of TA systems to induce cell lysis or cell stasis has been linked to biofilm and persister cell formation in pathogens [98], [129]. RelE for example, is a global inhibitor of translation during nutrient stress, and its expression reduces the chance of starvation by lowering the cell's nutrient requirements [130]. Doc, which appears to be quite conserved amongst strains as indicated by PCR (Fig. S1) and expressed under standard conditions, resembles members of another family of bacterial proteins called Fic. Bacterial toxin–antitoxin (TA) systems typically facilitate cell survival during intervals of stress allowing cells to switch to a reversible quasidormant state [131] and expression of such genes under standard conditions may be reflective of a subset of older or stressed cells in the culture. It is likely that these systems may be of particular importance relating to the viable but non-cultivable (VBNC) phenomenon observed within several *Campylobacter* species [15], [132].

## Conclusion

The pathogenic mechanisms responsible for acute intestinal infections by *Campylobacter* spp, although still poorly understood, are thought to involve adherence, cellular invasion, and toxin production. With the aid of whole genome analysis, comparative bioinformatics and secretome prediction we have identified a minimum of 106 potential virulence related factors, encompassing each of the known virulence tactics of pathogenic *Campylobacter* spp. Furthermore, similar to the emerging pathogen *C. concisus*, using genome comparisons and proteins profiles we propose the possibility of genomospecies within *C. ureolyticus*; a taxonomic continuum comprised of several species that are likely to have different impacts on human health and disease. *Campylobacter* species tend to be specialists not generalists, thus the presence of such a diverse number of virulence homologs warrants functional investigation. This study provides the first whole genome analysis of *C. ureolyticus* and a catalogue for the investigation and confirmation of this pathogen's virulence gene arsenal.

## Supporting Information

Figure S1 PCR analyses of the RTX 2861 bp (A), VirD4 1,625 bp (B), CiaB 1469 bp (C) and Doc 311 bp (D) genes in seven *Campylobacter ureolyticus* strains. Lane 1: Molecular weight marker Hyperladder I (A–C), Hyperladder II (D), lane 2: CIT01, lane 3: CIT02, lane 4: CIT04, lane 5: CIT 05, lane 6: CIT07, lane 7: CIT09, lane 8: DSM 20703 and lane 9: negative control. It is important to note that the absence of an amplicon does not confirm the absence of the gene. The primers are specific to the nucleotide sequences of *C. ureolyticus* DSM 20703 and/or *C. ureolyticus* ACS-301-V-Sch3b and does not account for single nucleotide polymorphisms or variable regions between strains within the primer annealing regions. This PCR should be seen as a confirmatory rather than exclusionary test. Also, rt-PCR indicated that of theses 4 genes, *doc* was the only protein expressed under standard conditions (in all 5 strains as above).
(TIF)

Table S1 Sequence based comparison: 128 protein coding genes of *C. ureolyticus* DSM 20703 and ACS-301-V-Sch3b highly conserved (>70% amino acid identity) across 8 different *Campylobacter* species and 1 subsp.
(XLSX)

Table S2 Sequence based comparison: 65 protein coding genes of *C. ureolyticus* DSM 20703 and ACS-301-V-Sch3b highly conserved within these strains (>70% amino acid identity) but no homologs (<25% amino acid identity) within 8 different *Campylobacter* species and 1 subspecies.
(XLSX)

Table S3 Sequence based comparison: 341 genes out of 1810 genes in *C. ureolyticus* DSM 20703 with below 25% identity in comparison to *C. ureolyticus* ACS-301-V-Sch3b.
(XLSX)

Table S4 Sequence based comparison: 290 genes out of 1700 genes in *C. ureolyticus* ACS-301-V-Sch3b with below 25% identity in comparison to *C. ureolyticus* DSM 20703.
(XLSX)

Table S5 Secretome of *C. ureolyticus* DSM 20703.
(XLSX)

Table S6 Secretome of *C. ureolyticus* ACS-301-V-Sch3b.
(XLSX)

Table S7 Sequence based comparison: 1469 out of 1810 genes in *C. ureolyticus* DSM 20703 and 1700 genes in *C. ureolyticus* ACS-301-V-Sch3b with greater than 25% identity.
(XLSX)

Table S8 Sequence based comparison: 1410 genes out of 1700 genes in *C. ureolyticus* ACS-301-V-Sch3b and 1810 genes *C. ureolyticus* DSM 20703 with greater than 25% identity.
(XLSX)
